# Time series-based hybrid ensemble learning model with multivariate multidimensional feature coding for DNA methylation prediction

**DOI:** 10.1186/s12864-023-09866-5

**Published:** 2023-12-11

**Authors:** Wu Yan, Li Tan, Li Mengshan, Zhou Weihong, Sheng Sheng, Wang Jun, Wu Fu-an

**Affiliations:** 1 School of Biotechnology, Jiangsu University of Science and Technology, Zhenjiang, Jiangsu 212018 China; 2 School of Mathematics and Computer Science, Gannan Normal University, Ganzhou, Jiangxi 341000 China; 3 Sericultural Research Institute, Chinese Academy of Agricultural Sciences, Zhenjiang, Jiangsu 212018 China; 4 College of Physics and Electronic Information, Gannan Normal University, Ganzhou, Jiangxi 341000 China

**Keywords:** DNA methylation, Time sequences, Feature coding, Ensemble learning

## Abstract

**Background:**

DNA methylation is a form of epigenetic modification that impacts gene expression without modifying the DNA sequence, thereby exerting control over gene function and cellular development. The prediction of DNA methylation is vital for understanding and exploring gene regulatory mechanisms. Currently, machine learning algorithms are primarily used for model construction. However, several challenges remain to be addressed, including limited prediction accuracy, constrained generalization capability, and insufficient learning capacity.

**Results:**

In response to the aforementioned challenges, this paper leverages the similarities between DNA sequences and time series to introduce a time series-based hybrid ensemble learning model, called Multi2-Con-CAPSO-LSTM. The model utilizes multivariate and multidimensional encoding approach, combining three types of time series encodings with three kinds of genetic feature encodings, resulting in a total of nine types of feature encoding matrices. Convolutional Neural Networks are utilized to extract features from DNA sequences, including temporal, positional, physicochemical, and genetic information, thereby creating a comprehensive feature matrix. The Long Short-Term Memory model is then optimized using the Chaotic Accelerated Particle Swarm Optimization algorithm for predicting DNA methylation.

**Conclusions:**

Through cross-validation experiments conducted on 17 species involving three types of DNA methylation (6 mA, 5hmC, and 4mC), the results demonstrate the robust predictive capabilities of the Multi2-Con-CAPSO-LSTM model in DNA methylation prediction across various types and species. Compared with other benchmark models, the Multi2-Con-CAPSO-LSTM model demonstrates significant advantages in sensitivity, specificity, accuracy, and correlation. The model proposed in this paper provides valuable insights and inspiration across various disciplines, including sequence alignment, genetic evolution, time series analysis, and structure–activity relationships.

## Background

**Fig. 1:**
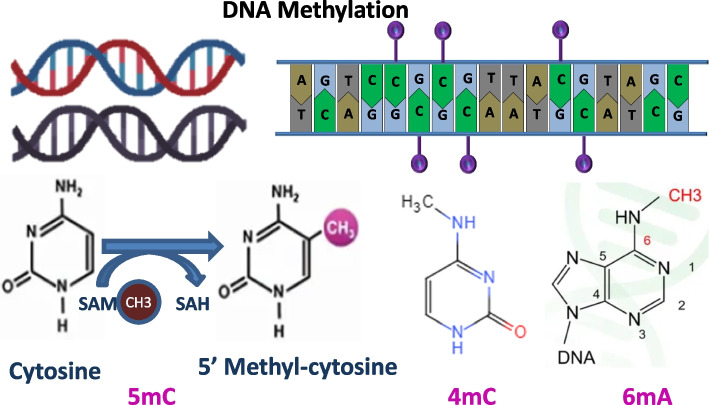
5mC, 6 mA, 4mC methylation composition and structure

DNA methylation is a form of DNA chemical modification, where a methyl group forms a covalent bond with the 5-carbon position of cytosine in CpG dinucleotides within the genome, under the catalysis of DNA methyltransferases [1–5]. This modification alters hereditary expression by modifying chromatin structure, DNA conformation and stability, and affecting DNA–protein interactions. Crucially, it regulates gene expression without modifying the DNA sequence itself. DNA methylation is influenced by a variety of factors, such as environmental conditions, climate, season, age, and diseases, which can lead to diverse activations, inductions, or suppressions of this modification [6–8]. The composition and structure of three types of methylation (5mC, 6 mA, and 4mC) are shown in Fig. 1. Research in DNA methylation primarily encompasses experimental detection techniques and theoretical computational models. Experimental approaches like bisulfite sequencing (WGBS) are resource-intensive and economically costly [9, 10]. Consequently, developing theoretical computational models for DNA methylation holds substantial research value and offers promising prospects for better understanding and studying gene regulatory mechanisms.

In recent years, models for predicting DNA methylation have attracted considerable attention [11–13]. Currently, machine learning and deep learning algorithms [14–19] are commonly used for model construction, such as random forest [20], fuzzy theory [21], decision tree [22], support vector machine [23], Bayesian method [24], convolutional neural network CNN [25–27], and long short-term memory network (LSTM) [28], and so on. Furthermore, a number of ensemble learning models [29–31] have been developed, incorporating advanced concepts like the attention mechanism [32–34] and Multi-Head Attention Mechanism [35, 36]. For instance, Li et al. [37] constructed a hybrid learning model combining LSTM and CNN for predicting DNA methylation sites, demonstrating impressive performance. Tsukiyama et al. [38], Liu et al. [39], Xu et al. [40] et al. proposed a series of predictive models [41, 42] that combine concepts from natural language processing, attention mechanism, and transfer learning, achieving promising results. Additionally, Lv et al. [43] proposed a hybrid framework called iDNA-MS for identifying DNA modification sites, employing random forests and three encoding methods. In parallel, Yu et al. [44, 45] also proposed two adaptive feature-based DNA methylation recognition methods, iDNA-AB and iDNA-ABT, which also exhibited good predictive capabilities.

Currently, machine learning algorithms are extensively utilized as the foundational theory in predicting DNA methylation sites, with particularly focus on methylation types such as 6 mA, 5mC, and 4mC [46–48], demonstrating impressive performance. The authors recognize that three outstanding issues remain to be explored and addressed: (1) The performance of machine learning-based DNA methylation models still needs further improvement. (2) Currently, most models are trained or tested only on a single type of DNA methylation, and their generalization ability needs to be improved. (3) Machine learning models have shortcomings in associating with the biological characteristics of the research problem itself, leading to poor feature extraction performance and consequently impacting the learning capability of the models. DNA sequences are comprised of character sequences constituted by four letters (ACGT), whereas time series are sequences of numbers or other symbols arranged in a chronological order. In terms of data representation, time series and biological sequences are essentially similar. In terms of mechanism, a time series is a sequence that progresses over time, whereas biological sequences represent gene relationships developed by organisms through the process of evolution over time.

In conclusion, from a theoretical perspective, the application of time series analysis methods in DNA sequence study has been demonstrated to be viable [49–52]. We attempt to propose feasible solutions to the aforementioned three problems. Firstly, considering the similarities between DNA sequences and time series, we propose a hybrid ensemble learning model, which employs time series methods to analyze the predictive performance of DNA methylation. Secondly, to assess the model’s generalization capacity across different species, we perform cross-species cross-validation experiments, focusing on various types of DNA methylation. Thirdly, we enhance the model’s learning potential by extracting multivariate features from DNA sequences and constructing a feature matrix for DNA sequences [53–58].

With these factors in mind, the paper proposes a multivariate multidimensional feature coding (Multi2) method, which combines three types of time series with three types of genetic features, resulting in nine encoding matrices. We then used CNN to extract features from the coding matrices, creating a feature matrix that includes DNA sequence timing, positional, physical–chemical, and biological information. Next, the parameters of the Long Short-Term Memory (LSTM) network were optimized using the Chaotic Accelerated Particle Swarm Optimization (CAPSO) algorithm [59], resulting in a hybrid ensemble learning model called Multi2-Con-CAPSO-LSTM. The model takes the feature matrix as input and predicts results of DNA methylation outcomes. The Multi2-Con-CAPSO-LSTM model is applied in predicting three different types of DNA methylation (6 mA, 5mC, and 4mC) across various species. Its performance is compared with six benchmark models to evaluate the overall performance of the Multi2-Con-CAPSO-LSTM model. The innovations and contributions of this study are as follows:

1. The utilization of multivariate encoding methods enhances the interpretability of the proposed model. The proposed encoding method (Multi2) merges temporal and genetic information from the DNA sequences into its features. A feature matrix is generated using the feature information from temporal context of time sequence and the physical, chemical, biological characteristics of the gene sequence.
2. The modeling strategy based on time series offers a new perspective for studying biological sequences. This strategy, which combines time series analysis methods with temporal encoding strategies, leverages the similarities between time series and biological sequences to provide new insights and references for modeling biological sequences.
3. The hybrid ensemble learning model exhibits promising prospects for both promotion and application. This model, integrating the strengths of CNN, CAPSO, and LSTM, has broad applications and substantial potential for advancement. It offers valuable insights for sequence research in fields like bioinformatics, evolutionary biology, and genetics, and provides crucial decision support across various research areas in disciplines such as engineering, computer science, chemistry, and biology.

## Methods

### Encoding representation of DNA sequences

A DNA sequence is denoted as $Seq={S}_{1}{S}_{2}\cdots {S}_{i}\cdots {S}_{n}$, ${S}_{i}\in \{A,C,T,G)$. This paper adopts three time series encoding methods [60–62] and three genetic feature encoding methods [63], combining them to form a multivariate multidimensional encoding representation for DNA sequences.

#### Time series encoding of DNA sequences

1. Coding of Spectral time Sequences

The time series representation of DNA sequences is denoted as $[{x}^{(1)},\cdots {x}^{(N)}]$. Spectral encoding obtains the time series using the Eq. (1).

1 $${x}^{(i)}=\left\{\begin{array}{llll} 1,&{S}_{i}=a&\\2,&{S}_{i}=g&\\3,&{S}_{i}=c,&i=\mathrm{1,2},\dots ,n\\4,&{S}_{i}=t&\end{array}\right.$$

Where ${\mathrm{x}}^{(\mathrm{ i})}$ represents the time sequence data at position $\mathrm{i}$, while ${\mathrm{S}}_{\mathrm{i}}$ corresponds to the DNA sequence data at the same position.

2 CGR time sequence

The four vertices of a square are representative of the four types of nucleotides in a DNA sequence. The position of the subsequent nucleotide is determined by utilizing the coordinates associated with each nucleotide.

- Step (1): The initial state of the vertices is established as follows: A(1, 1)、T(-1, -1)、G(-1, 1)、C(1, -1);
- Step (2): The center point (0, 0) is designated as the initial position;
- Step (3): Beginning with the first nucleotide, plot a point at the midpoint between its corresponding vertex and the center point (0, 0);
- Step (4): Taking the second character as the current character, plot a point at the midpoint between its corresponding vertex and the point representing the previous character.
- Step (5): Proceed to the next nucleotide as the current character and continue repeating Steps (4) and (5) until the DNA sequence is fully represented, as detailed in Eq. (2).
  2 $$\begin{array}{c}{x}^{\left(i\right)}={CGR}_{i}={CGR}_{i-1}-\frac{{CGR}_{i-1}-{\mathrm{g}}_{i}}{2}\\ {\mathrm{g}}_{i}=\left\{\begin{array}{llll}\left( 1 , 1 \right),{S}_{i}=a\\ \left( -1, 1 \right),{S}_{i}=g\\ \left( 1,-1 \right),{S}_{i}=c\\ \left(-1,-1\right),{S}_{i}=t\end{array}\right.\end{array}$$

Where ${\mathrm{CGR}}_{i}$ represents the $\mathrm{CGR}$ time sequence at the $i$-th iteration, and ${\mathrm{g}}_{i}$ corresponds to the DNA sequence data at position $i$.

3 Z time sequence

In the DNA sequence, the number of occurrences of $\mathrm{A}$, $C$, $\mathrm{G}$ and $\mathrm{T}$ up to the $i$-th base are denoted as ${A}_{i},{C}_{i},{G}_{i},{T}_{i}$,respectively. The $Z$ time sequence is defined as Eq. (3).

3 $$\begin{array}{c}{x}^{(i)}=\sqrt{{X}_{i}+{Y}_{i}+{Z}_{i}}\\ \left\{\begin{array}{c}{X}_{i}=\left({A}_{i}+{G}_{i}\right)-({C}_{i}+{T}_{i})\\ {Y}_{i}=\left({A}_{i}+{C}_{i}\right)-({G}_{i}+{T}_{i})\\ {Z}_{i}=\left({A}_{i}+{T}_{i}\right)-({C}_{i}+{G}_{i})\end{array}\right.\end{array}$$

Where ${X}_{i},{Y}_{i}{,Z}_{i}$ represent the coordinate values of $X$-axis, $Y$-axis, $Z$-axisi.

#### Gene feature encoding for DNA sequences

1. Binary encoding of Position Feature (BPF)

The BPF is a sparse binary four-dimensional vector [64], and its encoding method is defined as Eq. (4).

4 $$b=\left\{\begin{array}{c}\left(1,0,0,0\right),\;if\;b=A\\\left(0,1,0,0\right),\;if\;b=T\\\left(0,0,1,0\right),\;if\;b=G\\\left(0,0,0,1\right),\;if\;b=C\end{array}\right.$$

The BPF encoding is transformed into a feature matrix of size $4\times L$ as shown in Eq. (5).

5 $${A}_{1}=\left[\begin{array}{cccc}{BPF}_{1}\left(1\right)&\cdots& {BPF}_{1}\left(L\right)\\\vdots&\ddots&\vdots\\{BPF}_{4}\left(1\right)&\cdots&{BPF}_{4}\left(L\right)\end{array}\right]$$

(2) Coding of Nucleic acid chemical properties (NCP)

NCP is a coding method based on hydrogen bonding strength, ring structure, and biological composition [41], as shown in Eq. (6).

6 $$\begin{array}{ccc}{NCP}_1\left(i\right)=\left\{\begin{array}{c}1\;if\;D_i\;\in\lbrack A,G\rbrack\\0\;if\;D_i\;\in\lbrack C,T\rbrack\end{array},\right.&{NCP}_2\left(i\right)=\left\{\begin{array}{c}1\;if\;D_i\;\in\lbrack A,T\rbrack\\0\;if\;D_i\;\in\lbrack C,G\rbrack\end{array}\right.,&{NCP}_3\left(i\right)=\left\{\begin{array}{c}1\;if\;D_i\;\in\lbrack A,C\rbrack\\0\;if\;D_i\;\in\lbrack G,T\rbrack\end{array}\right.\end{array}$$

In a DNA sequence of length $L$, the NCP encoding will generate a feature matrix of size $3\times L$, as shown in Eq. (7).

7 $${A}_{2}=\left[\begin{array}{ccc}{NCP}_{1}\left(1\right)&\cdots&{NCP}_{1}\left(L\right)\\\vdots&\ddots&\vdots\\{NCP}_{3}\left(1\right)&\cdots&{NCP}_{3}\left(L\right)\end{array}\right]$$

(3) Coding of Dinucleotide physical and chemical properties (DPCP)

The DPCP encoding encompasses angle change parameters for adjacent base spatial planes in the vertical, forward–backward, and left–right directions. It also includes distance change parameters for the relative positions of adjacent bases in these directions. Then, these parameters are normalized using a specific method detailed in Eq. (8):

8 $${X}_{n}= \frac{X- {X}_{min}}{{X}_{max}- {X}_{min}}$$

To match the column dimensions of other encoding schemes, a sliding dipeptide window algorithm is used to calculate the DPCP values, as shown in Eq. (9).

9 $${DPCP}_{n}\left(i\right)= \frac{{X}_{n}\left({D}_{i-1}{D}_{I}\right)+{X}_{n}({D}_{i}{D}_{i-1})}{2}$$

${DPCP}_{n}\left(i\right)$ represents the $i$-th physical and chemical property of the $n$-th nucleotide, while ${X}_{n}$ represents the physicochemical property of the $n$-th nucleotide. Through calculations, a feature matrix of size $6\times L$ is obtained, as shown in Eq. (10).

10 $${A}_{3}=\left[\begin{array}{ccc}{DPCP}_{1}\left(1\right) &\cdots &{DPCP}_{1}\left(L\right)\\\vdots&\ddots&\vdots\\{DPCP}_{6}\left(1\right)&\cdots&{DPCP}_{6}\left(L\right)\end{array}\right]$$

#### Multivariate multidimensional encoding representation of DNA sequences

By combining the three types of time series encoding and three types of genetic feature encoding mentioned above, we have derived a total of nine encoding methods for DNA sequence representation. This array includes six single encodings and three hybrid encodings, with the details provided in Table 1.

**Table1:** Encoding representation of DNA sequences

Symbol	Abbreviation	Description
Code 1	Spectral encoding	Spectral time sequence
Code 2	CGR encoding	CGR time sequence
Code 3	Z encoding	Z time sequence
Code 4	Time sequence encoding	Spectral time sequence + CGR time sequence + Z time sequence
Code 5	BPF encoding	Binary encoding of Position Feature
Code 6	NCP encoding	Coding of Nucleic acid chemical properties
Code 7	DPCP encoding	Coding of Dinucleotide physical and chemical properties
Code 8	Gene feature encoding	Binary encoding of Position Feature + Nucleic acid chemical properties + Coding of Dinucleotide physical and chemical properties
Code 9	Multidimensional and multivariate hybrid encoding	Hybrid Time sequence encoding and Gene feature encoding

Time series encoding methods can capture the contextual relationships within DNA sequences, whereas gene feature encoding reflects the physical, chemical, or biological information inherent to the DNA sequences. In Table 1, Code 4 denotes the mixed encoding of time series, Code 8 represents the mixed encoding of gene features, and Code 9 signifies the combined encoding of both time series and gene features.

### Model framework

**Fig. 2:**
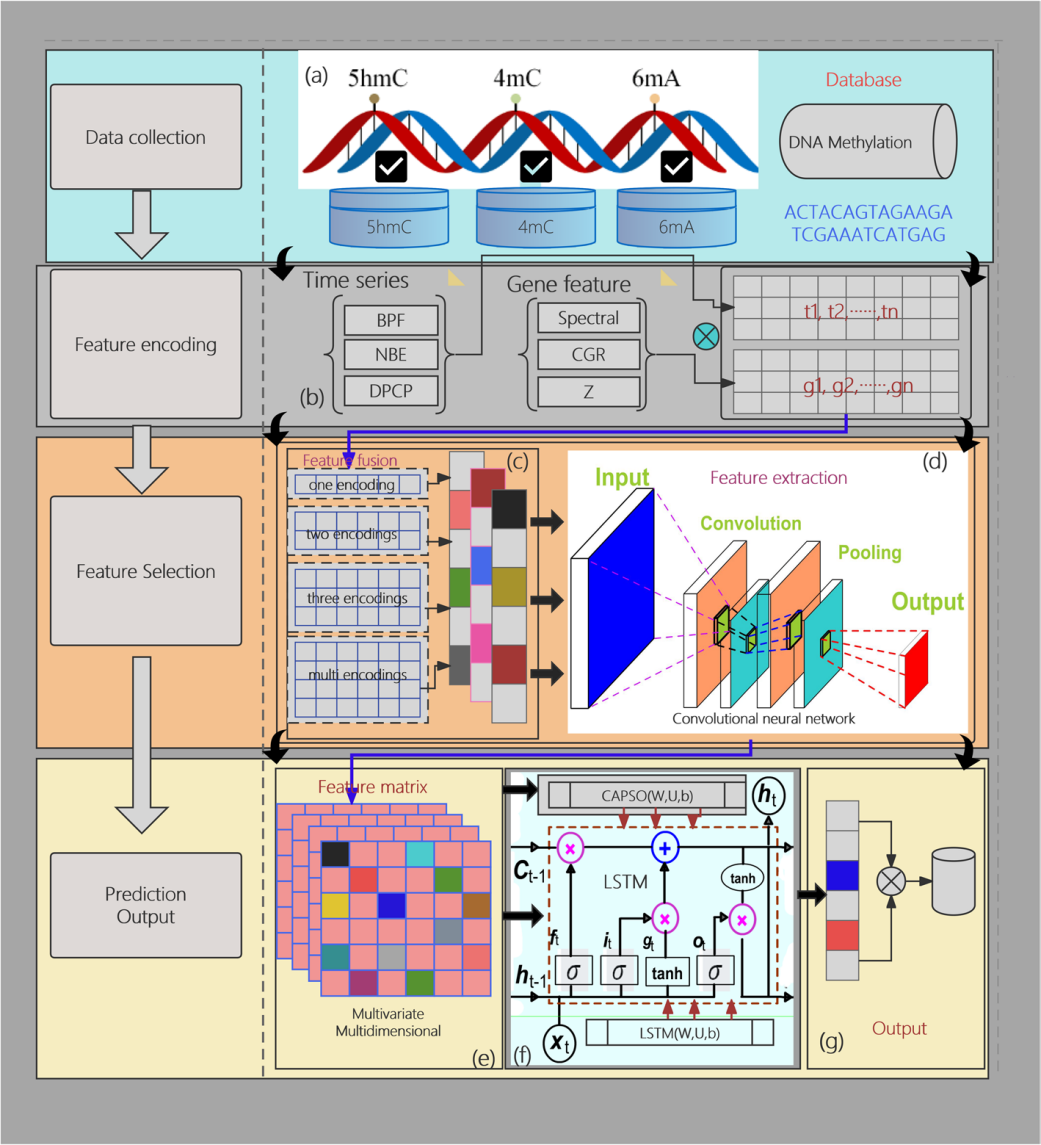
Overview of the proposed model. **a** DNA data collection **b** Feature encoding **c** Feature fusion **d** Feature information extraction **e** Feature information matrix **f** CAPSO-LSTM modeling **g** The output

The Multi2-Con-CAPSO-LSTM model comprises of four stages: data collection, feature encoding, feature selection, and modeling and prediction, as depicted in Fig. 2. The first stage is the data collection phase. The collected data is organized to construct a dataset of methylation sites. The second stage is the feature encoding stage. The methylation data is processed using time series encoding and gene feature encoding methods (with three encoding methods for each), resulting in a multivariate multidimensional encoded sequence. The third stage is the feature selection phase, during which the various encoding sequences are fused to construct a two-dimensional encoding matrix. This study employs CNN for feature extraction, selecting encoding features to compose the final feature matrix. The final stage, modeling and prediction, involves modeling the feature matrix and inputting it into the LSTM model for training. The parameters of LSTM are optimized using the CAPSO algorithm [65]. After training and validation of the model, we conducted testing and obtained DNA methylation prediction data as the output of the model.

The implementation steps of the model are as follows:

- Step 1: Data Collection and Preprocessing. The collected data is preprocessed by removing duplicate entries to obtain the training set, validation set, and test set, forming the experimental dataset.
- Step 2: Data Encoding and Fusion. Utilize three types of time series encoding and three types of genetic feature encoding to respectively code the data in the dataset, and then fuse these time series and genetic feature encodings, ultimately resulting in a total of nine combined encoding methods.
- Step 3: Feature Extraction. Using convolutional neural networks to extract features from encoded sequences, characteristic information from various encoded sequences is effectively captured.
- Step 4: Feature Selection and Feature Matrix Generation. Feature selection is performed on the features extracted from sequences with various encodings. The selected features are then compiled into a feature matrix, resulting in a multivariate multidimensional data feature matrix.
- Step 5: LSTM Model Construction and Parameter Optimization. The multivariate multidimensional feature matrix is used as the input to construct and train the LSTM model, setting relevant parameters. For detailed information on the model construction, please refer to “Model construction” section.
- Step 6: Model Validation. Validate the model with the validation dataset by looping through steps 2 to 5, optimizing model parameters to minimize output errors.
- Step 7: Model Testing and Output. Utilize the test dataset to assess the model by looping through steps 2 to 5, and produce the test results.
- Step 8: Output. Perform statistical analysis on each output result.

### Model construction

The Multi2-Con-CAPSO-LSTM model takes $N$ DNA sequences $[{x}^{(1)},\cdots {x}^{(N)}]$ as input and generates a multivariate multidimensional encoding matrix following the fusion of different encodings [66, 67]. The DNA encoding sequence ${x}_{t}$ is defined by Eq. (11).

11 $${x}_{t}=Merge(funi\left({x}_{t-T:t-1}\right), fmulti\left({x}_{t-T:t-1}\right))$$

Where, ${x}_{t-T}$ represents the sequence of length T; $Merge(\bullet )$ is a fusion function that fuses the time series encoding $funi(\bullet )$ and the gene sequence encoding $fmulti(\bullet )$.

The multivariate multidimensional encoding matrix is convolved using CNN, as shown in Eq. (12), to obtain the feature matrix.

12 $${C}^{(k)}={W}^{(k)}\times {X}_{t-T:t-1}$$

Where, ${C}^{(k)}$ represents the convolution result, and ${W}^{(k)}$ represents the $k$-th convolution kernel.

**Fig. 3:**
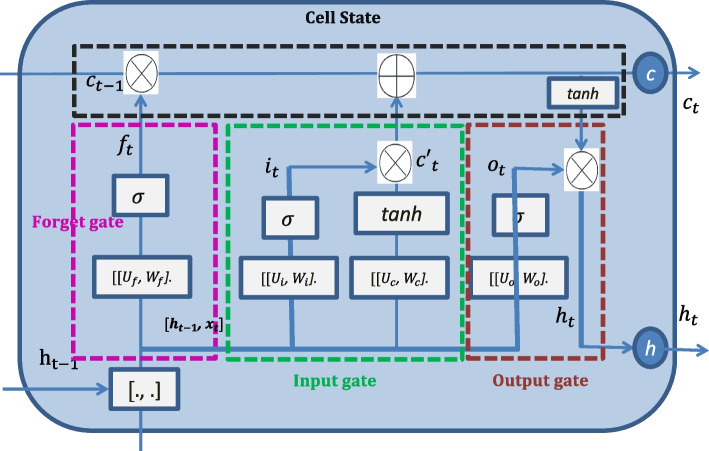
The unit of long short-term memory

After completing the convolutional feature extraction, the obtained feature matrix serves as the input to the LSTM model. The LSTM model consists of Gate Units and Memory Units, as shown in Fig. 3.

LSTM modeling involves four steps:

1. By computing the forget gate, the forget factor can be obtained, as shown in Eq. (13).

13 $${\mathrm{f}}_{t}=\upsigma ({\mathrm{W}}_{f}{\mathrm{x}}_{t}+{\mathrm{U}}_{f}{\mathrm{h}}_{t-1}+{\mathrm{b}}_{f})$$

Where, ${\mathrm{f}}_{t}$ represents the forget factor; $\upsigma (\bullet )$ represents the sigmoid activation function, which maps values to the range [0,1]; $({\mathrm{W}}_{f},{\mathrm{U}}_{f},{\mathrm{b}}_{f})$ represents the weight factors of the forget gate.

(2) By calculating the input gate, the input factor can be obtained, as shown in Eq. (14). Simultaneously, a new cell state is generated.

14 $${i}_{t}=\upsigma ({\mathrm{W}}_{i}{\mathrm{x}}_{t}+{\mathrm{U}}_{i}{\mathrm{h}}_{t-1}+{\mathrm{b}}_{i})$$

Where, ${i}_{t}$ represents the input factor with a value range of [0,1], and $({\mathrm{W}}_{i},{\mathrm{U}}_{i},{\mathrm{b}}_{i})$ represents the weight factors of the input gate. After obtaining the input factor, LSTM creates a new cell state using Eq. (15).

15 $${\mathrm{C}}_{t}{\prime}=\mathrm{tanh}({\mathrm{W}}_{c}{\mathrm{x}}_{t}+{\mathrm{U}}_{c}{\mathrm{h}}_{t-1}+{\mathrm{b}}_{c})$$

Where, ${\mathrm{C}}_{t}{\prime}$ represents the new cell state;$\mathrm{tanh}(\bullet )$ is an activation function with a range of [-1,1], and $({\mathrm{W}}_{c},{\mathrm{U}}_{c},{\mathrm{b}}_{c})$ are the weight factors used to compute the new cell state.

(3) As shown in Eq. (16), the cell state is updated.

16 $${{\mathrm{C}}_{t}={\mathrm{f}}_{t}*{\mathrm{C}}_{t-1}+{i}_{t}*\mathrm{C}}_{t}{\prime}$$

(4) The output factor is calculated as shown in Eq. (17).

17 $${\mathrm{o}}_{t}=\upsigma ({\mathrm{W}}_{o}{\mathrm{x}}_{t}+{\mathrm{U}}_{o}{\mathrm{h}}_{t-1}+{\mathrm{b}}_{o})$$

Where, the output factor ${\mathrm{o}}_{t}$ determines the current output or the input for the next state; $({\mathrm{W}}_{o},{\mathrm{U}}_{o},{\mathrm{b}}_{o})$ represents the weight factors of the output gate.

The LSTM model has three sets of weight factors, denoted as $(\mathrm{W},\mathrm{U},\mathrm{b})$. In this study, the parameters are optimized using the CAPSO algorithm. Unlike traditional PSO, CAPSO utilizes only the exploration factor during the optimization process [59], and the particle iteration is defined as shown in Eq. (18):

18 $${\mathrm{x}}_{i,d}^{k+1}={(1-\mathrm{C}}_{2}){\mathrm{x}}_{i,d}^{k}+{\mathrm{C}}_{2}{\mathrm{p}}_{g,d}^{k}+{\mathrm{C}}_{2}r$$

Where, ${\mathrm{x}}_{i,d }^{k}$ represents the position of particle $i$ in dimension d at the $k$-th iteration, and $r$ is a random number. ${C}_{2}$ is obtained from the chaotic variable generated by the Logistic equation, as shown in Eq. (19).

19 $${\mathrm{d}}_{i}^{k+1}=4{\mathrm{d}}_{i}^{k}(1-{\mathrm{d}}_{i}^{k})$$

When $0<{\mathrm{d}}_{i}^{k}<1$, the resulting ${C}_{2}$ is in a fully chaotic state.

When applying the CAPSO algorithm to optimize the $(\mathrm{W},\mathrm{U},\mathrm{b})$ parameters, the particle structure is defined as shown in Eq. (20).

20 $${y}_{pso}={f}_{\mathrm{ca}pso}(W,U,b)$$

Finally, the output is obtained by multiplying the output factor with the cell state, as depicted in Eq. (21).

21 $${h_t=o}_t\ast tanh\;(C_t)$$

## Experiments

### Experimental data

The dataset utilized in this study was obtained from a benchmark dataset [43], encompassing three types of DNA methylation from 17 different species, amounting to a total of 30,4619 records. According to different species, we derived 17 sub-datasets. Each sub-dataset was divided into training set, validation set, and test set, with proportions of 70%, 15%, and 15%, respectively. Data statistics are shown in Table 2.

**Table 2:** Experimental data distribution

Dataset	Species	Type	Training(70%)	Validation(15%)	Testing(15%)	Total
1	H.sapiens	5hmC	2915	624	624	4163
2	M.musculus	5hmC	5152	1103	1103	7358
3	C.equisetifolia	4mC	2772	593	593	3958
4	F.vesca	4mC	22116	4739	4739	31594
5	S.cerevisiae	4mC	2772	593	593	3958
6	Tolypocladium	4mC	21456	4598	4598	30,652
7	D.melanogaster	6 mA	15668	3357	3357	22382
8	R.chinensis	6 mA	838	180	180	1198
9	Xoc BLS256	6 mA	24102	5164	5164	34430
10	C.elegans	6 mA	11146	2388	2388	15922
11	T.thermophile	6 mA	11146	2388	2388	15922
12	A.thaliana	6 mA	44622	9562	9562	63746
13	H.sapiens	6 mA	25670	5500	5500	36670
14	C.equisetifolia	6 mA	8492	1820	1820	12132
15	F.vesca	6 mA	4344	930	930	6204
16	S.cerevisiae	6 mA	5300	1136	1136	7572
17	Tolypocladium	6 mA	4730	1014	1014	6758
Total			213241	45689	45689	304619

### Model evaluation metrics

This study employs five commonly used evaluation metrics, namely sensitivity (SN) reflecting true positive rate, specificity (SP) reflecting true negative rate, accuracy (ACC), Matthews correlation coefficient (MCC) reflecting correlation, and Area Under ROC Curve (AUC). Their definitions are shown in Eq. (22):

22 $$\left\{\begin{array}{c}SN=\frac{TP}{TP+FN} \times 100\%\\ SP=\frac{TN}{TN+FP} \times 100\%\\ ACC=\frac{TP+TN}{TP+FN+TN+FP} \times 100\%\\ MCC=\frac{TP\times TN-FP\times FN}{\sqrt{\left(TP+FP\right)\times \left(TP+FN\right)\times \left(TN+FP\right)\times (TN+FN)}} \\ AUC=\frac{\sum_{i\in pos}{rank}_{i}-\frac{{num}_{pos}({num}_{pos}+1)}{2}}{{num}_{pos}{num}_{neg}}\end{array}\right.$$

Where TP, TN, FN, and FP represent true positives, true negatives, false negatives, and false positives, respectively.

## Results and discussion

### Experimental results

**Fig. 4:**
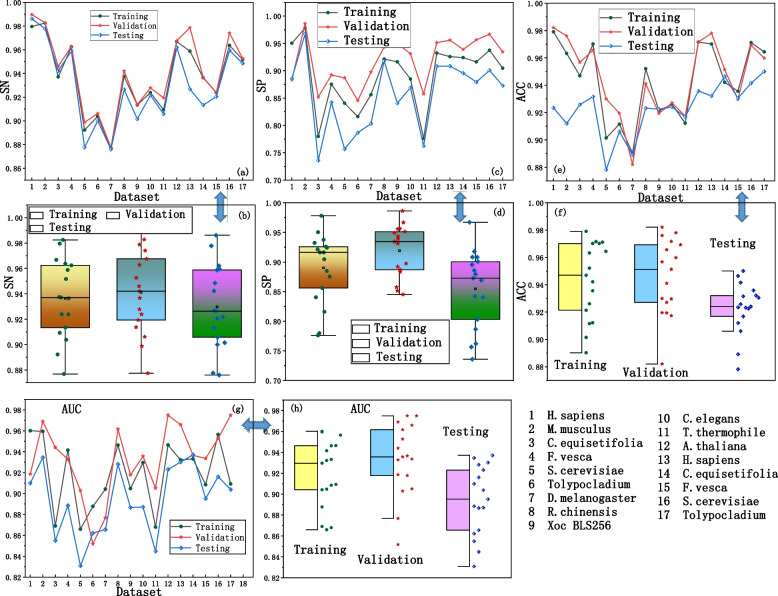
The prediction result curves and statistical results of the model on the training set, validation set, and testing set. **a** SN curve. **b** SN distribution statistics. **c** SP curve. **d** SP distribution statistics. **e** ACC curve. **f** ACC distribution statistics. **g** AUC curve. **h** AUC distribution statistics

We trained and validated the Multi2 Con CAPSO LSTM using 17 sets of training and validation data. Through adjustments of various parameters, our goal was to minimize the model's error. Then, we finally apply the model to the testing data. The prediction results for the training set, validation set, and testing set are shown in Fig. 4.

By observing the positions of the result curves, it can be observed that the validation set curves are situated at the top, indicating the best predictive performance, as shown in Fig. 4a, c, e, g. The training set curve is slightly lower, while the testing set curve is positioned at the bottom, suggesting a decreasing predictive performance from the validation set to the training set and then to the testing set. Upon analyzing the data distribution and statistics of the predicted results, it becomes evident that the validation set demonstrates the best performance, indicating that the model has been sufficiently trained. The average results of the model on each dataset are shown in Table 3.

**Table 3:** The averages predicted results on the training set, validation set, and test set

Index	Training	Validation	Testing
SN	0.9386	0.9388	0.9350
SP	0.8903	0.9157	0.8748
ACC	0.9429	0.9457	0.9229
MCC	0.9068	0.9243	0.9027
AUC	0.9189	0.9326	0.8940

Table 3 also demonstrates that the validation set exhibits superior prediction performance. The training set is used to train the model's parameters, while the validation set is used to optimize these parameters based on the training. Therefore, it is anticipated that the validation set would display better overall performance. The testing set is used to evaluate the model's generalization ability with new samples. As a result, the overall predictive capability of the model on the test set is not as good as on the training and validation sets. However, the average metric values on the testing set are also around 0.9, indicating that the Multi2-Con-CAPSO-LSTM model possesses robust overall predictive capability and strong generalization capability.

### Discussion of different DNA methylation types

**Fig. 5:**
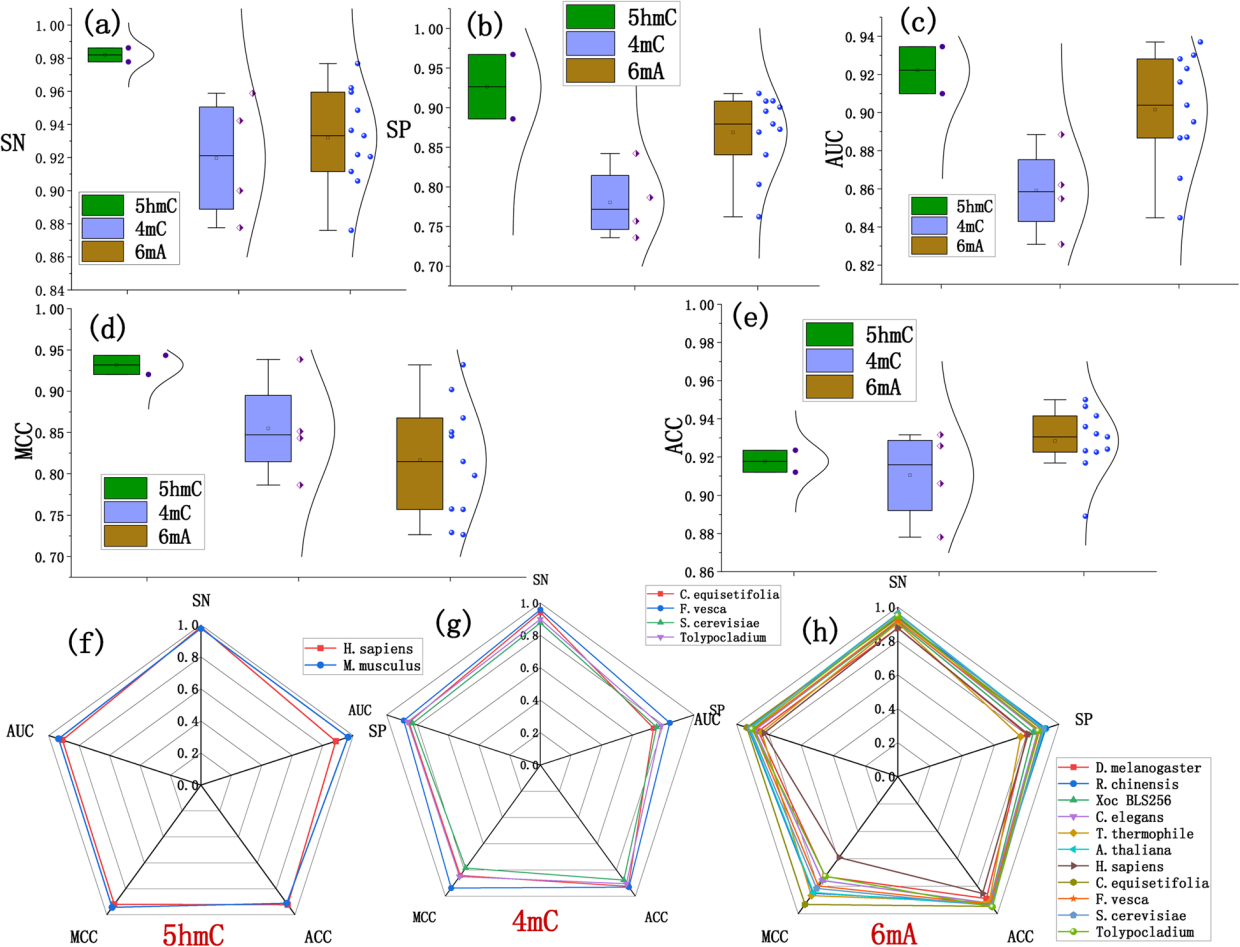
The performance of the model in 5hmC, 4mC and 6 mA. **a** ~ **e** show the data distribution and statistical results of SN, SP, ACC, MCC, and AUC for different species and different types of DNA methylation. **f** ~ **h** display the radar graphs for each evaluation metric

To evaluate the model's predictive performance across different DNA methylation types, the model's performance metrics for each DNA methylation type (5hmC, 4mC, and 6 mA) in 17 species are plotted in Fig. 5.

From the data distribution and statistical graphs, it can be observed that sensitivity, accuracy, and AUC are all above 0.82, with an average distribution around 0.92. The SP and MCC are also exceed 0.70, with an average around 0.85. All these metrics indicate that the model performs well for all types of DNA methylation, especially in the 5hmC test subset, where the SN reaches 0.97, and SP, ACC, MCC, and AUC are all above 0.9.

The results indicate that the Multi2-Con-CAPSO-LSTM shows slightly different predictive performance among different DNA methylation types. The performance of this model is quite consistent, and the model can effectively predict various methylation types. The average testing results for different DNA methylation types are shown in Table 4.

**Table 4:** The testing results of 5hmC, 4mC and 6 mA

Index	5hmC	4mC	6 mA	Average
SN	0.9820	0.9197	0.9320	0.9445
SP	0.9265	0.7804	0.8689	0.8586
ACC	0.9177	0.9104	0.9284	0.9188
MCC	0.9319	0.8548	0.8164	0.8677
AUC	0.9222	0.8591	0.9016	0.8943

Through comprehensive analysis of experimental results for three different DNA methylation types (5hmC, 4mC, and 6 mA), it can be observed that the Multi2-Con-CAPSO-LSTM can effectively predict these DNA methylation types. These results further demonstrate that the Multi2-Con-CAPSO-LSTM exhibits exceptional performance in predicting multi-types of DNA methylation. From the experiments involving the three DNA methylation types, Multi2-Con-CAPSO-LSTM can predict both single type of DNA methylation and multi-type of DNA methylation.

### Discussion of different feature encodings

**Fig. 6:**
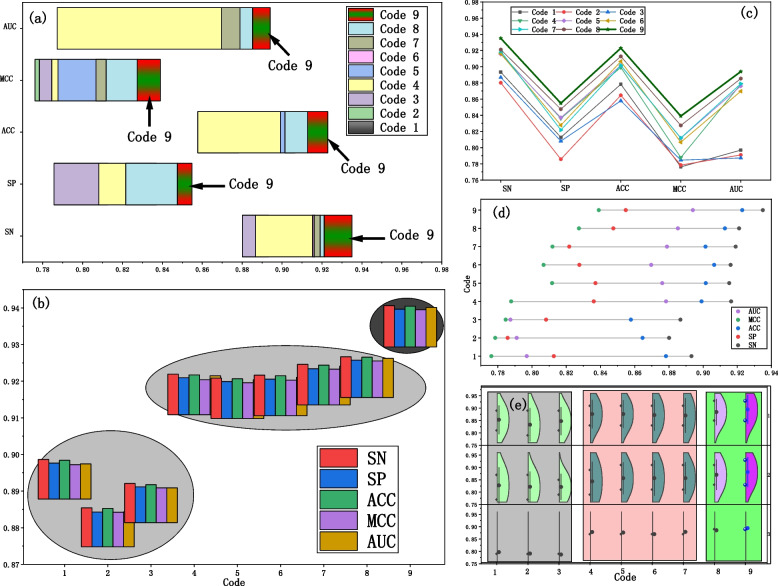
The prediction results for different encoding methods. **a** Floating histogram. **b** Bar chart. **c** Dot plot. **d** Scatter plot. **e** Box plot

The study employs six different DNA sequence encoding methods, resulting in a total of nine encoding representations. We have two primary concerns: Firstly, Does the utilization of different encoding methods directly impact the model's performance? Secondly, is the hybrid encoding approach more effective? To address these questions, we conducted experiments employing six individual encoding methods and three hybrid encoding approaches. The prediction results based on each encoding method are shown in Fig. 6.

The hybrid encoding approach (code 9) that combines time series and genetic features demonstrates excellent performance across various evaluation metrics, showing significant advantages in terms of true positive rate, true negative rate, and accuracy, as shown in Fig. 6a, c, d. Based on the observations from Fig. 6b and e, the following conclusions can be drawn: The model performance is the weakest when using a single time series encoding method (code 1, code 2, code 3); The performance of the model using a hybrid time series encoding method (code 4) is comparable to that of using a single genetic feature encoding method (code 5, code 6, code 7); The model performance using a hybrid genetic feature encoding method (code 8) is slightly superior to that of using a single genetic feature encoding method; The hybrid encoding method that combines time series and genetic features (code 9) demonstrates overall good performance, with all five evaluation metrics values exceeding 0.9.

Among different encoding methods, the single time series encoding (code 1, code 2, code 3) includes time-related feature information. However, because DNA methylation sites are only related to features within their extremely small window, the information extracted from these encodings is insufficient, thereby impacting the model's performance. Single gene feature encoding (code 5, code 6, code 7) includes information about the position, physicochemical properties, and biological aspects of the gene sequence. During the modeling process, relevant features are extracted from single gene feature encoding, resulting in an improvement in the predictive performance of the model. Similarly, code 9 effectively fuses the temporal information from the time series and the positional information, physicochemical properties, and biological information of gene features. Code 9 demonstrates an advantage in feature extraction by capturing more intrinsic correlated information within the DNA sequences, which ensures the model's performance.

Through various feature encoding experiments, it is demonstrated that we have answers to both of the questions of concern. Firstly, the nine encoding methods directly impact the model's performance. Secondly, the hybrid encoding methods (Code 4, Code 8 and Code 9) have significant performance advantages. Especially, the multidimensional multivariate hybrid encoding (Code 9) not only considers the pre and post sequence correlation of DNA methylation, but also incorporates the positional information, physiological properties, and biological information of the DNA sequence. As a machine learning model, Multi2-Con-CAPSO-LSTM can fuse these DNA methylation features, which ensures good performance.

### Discussion of different species

**Fig. 7:**
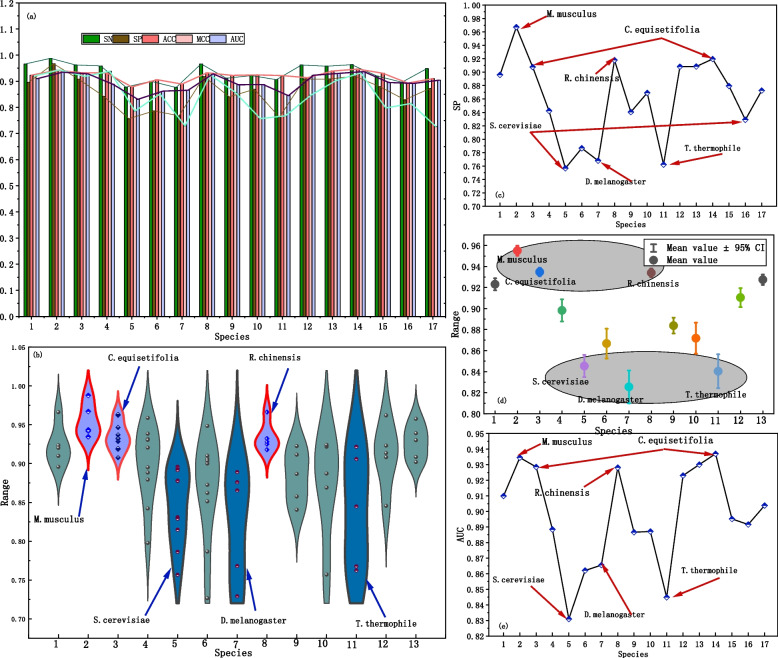
Predictive Results for Different Species. **a** Bar chart of various evaluation index **b** Box plot of different species. **c** Dot-line plot of SN. **d** Interval plot. **e** Dot-line plot of AUC

To evaluate the predictive performance of Multi2-Con-CAPSO-LSTM and analyze the performance variations among different species, we conducted a statistical analysis of the predictive evaluation metrics for the model across 17 different species. The results are presented in Fig. 7.

It can be observed that species 2, species 3, and species 14 demonstrate relatively good predictive performance, while species 5, species 7, and species 11 show slightly poorer predictive results, as shown in Fig. 7a, c, e. As shown in Fig. 7b and d, the data distribution for the three species, M.musculus, C.equisetifolia, and R.chinensis, is concentrated in the upper region, indicating better predictive performance. However, the data distribution of S. cerevisiae, D. melanogaster, and T. thermophile, is concentrated in the lower region, indicating slightly poorer predictive performance for these species. Table 5 shows the performance of the model of different species.

**Table 5:** The performance for different species

Species	SN	SP	ACC	MCC	AUC
H.sapiens	0.9662	0.8958	0.9235	0.9203	0.9099
M.musculus	0.9878	0.9672	0.9420	0.9435	0.9346
C.equisetifolia-4mc	0.9622	0.9076	0.9326	0.9184	0.9286
F.vesca	0.9588	0.8422	0.9316	0.9385	0.8885
S.cerevisiae	0.8776	0.7567	0.8781	0.7863	0.8309
Tolypocladium	0.9000	0.7866	0.9061	0.8513	0.8621
D.melanogaster	0.8760	0.7680	0.8891	0.7291	0.8655
R.chinensis	0.9664	0.9179	0.9323	0.9251	0.9281
Xoc BLS256	0.9115	0.8405	0.9225	0.8576	0.8867
C.elegans	0.9217	0.8691	0.9241	0.7570	0.8871
T.thermophile	0.9058	0.7623	0.9217	0.7676	0.8448
A.thaliana	0.9620	0.9082	0.9136	0.8456	0.9230
H.sapiens	0.9577	0.9084	0.9391	0.9020	0.9301
C.equisetifolia-6 mA	0.9633	0.9195	0.9465	0.9319	0.9370
F.vesca	0.9205	0.8793	0.9305	0.7977	0.8952
S.cerevisiae	0.8960	0.8290	0.8929	0.8148	0.8916
Tolypocladium	0.9485	0.8726	0.9101	0.7265	0.9038

The statistical results in Table 6 also indicate that the evaluation indicators of the three species, M.musculus, C.equisetifolia, and R.chinensis, are all above 0.9, indicating good performance. Where S. cerevisiae, D. melanogaster, and T. thermophile, the most evaluation indicators are distributed between 0.81 and 0.90. From the overall predictive results, the model's predictive performance exhibits slight variation among different species, but it can still effectively predict the methylation status in each species.

**Table 6:** Benchmark models in this paper

Model	Model details	References
iDNA-MS	Random Forest algorithm http://lin-group.cn/server/iDNA-MS	Lv, ect. 2020 [43]
iDNA–ABT	Adaptive embedding based on Bidirectional Encoder Representations from Transformers together with transductive information maximization	Yu, ect. 2021 [45]
iDNA-AB	iDNA-ABT using the cross-entropy loss	Yu, ect. 2021 [45]
EA-LSTM	Evolutionary attention-based LSTM	Li, etc. 2019 [68]
CTS-LSTM	LSTM network for correlated time series	Wan, etc. 2020 [69]
Conv-LSTM	Convolutional neural network and LSTM	Fu, etc. 2022 [70]

### Discussion of cross-validation under cross-species

**Fig. 8:**
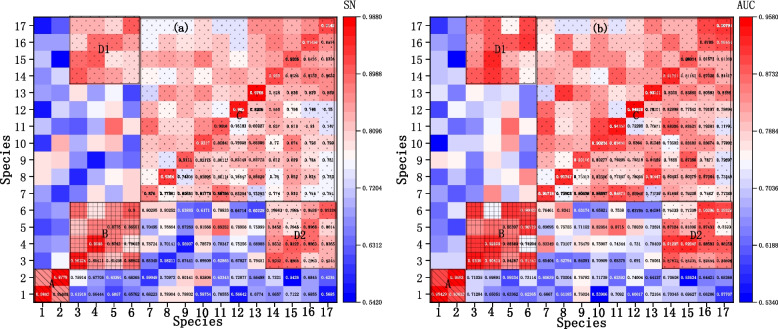
Heatmap of Cross-validation under cross-species. **a** Sensitivity (SN). **b** Area Under the Curve (AUC). (The x-axis represents the training species, and the y-axis represents the testing species.)

To investigate the model's generalization ability and validate its performance in predicting methylation in other species, we conducted cross-species validation experiments using different species in both the training and testing sets. Firstly, one species is selected from the dataset of 17 species for model training. Next, we conducted testing of the model using the remaining 16 species (excluding the one used for training). The heatmaps of SN and AUC for the cross-validation of each species are shown in Fig. 8.

The main diagonal blocks indicate that under the same species, the model demonstrates excellent predictive performance, with most SN and AUC values above 0.9, and a few slightly below 0.9, as shown in Fig. 8. It can be observed that the testing performance is good between Specie 1 and Specie 2. Similarly, the performance is also good among Specie 3 to Specie 6. Additionally, the performance among the 11 species from Specie 7 to Specie 17 is favorable as well. These results demonstrate high sensitivity and AUC values, all exceeding 0.8. Moreover, the predictive performance among Specie 3 to Specie 6 and Specie 14 to Specie 17 also shows good performance, with SN and AUC values mostly ranging between [0.8, 0.9].

The cross-species validation experiments show that the model performs best when trained and tested on the same species. Additionally, the model demonstrates excellent performance across different species with the same methylation type, as well as within the same species with different methylation types. The performance of the model is relatively satisfactory in different methylation types and different species. The above results indicate that Multi2-Con-CAPSO-LSTM exhibits good generalization ability and scalability.

### Discussion with other benchmark models

We selected two categories of models as benchmarking comparisons. The first category consists of general methylation predictors, while the second category encompasses enhanced prediction methods based on LSTM. In total, there are six models participating in the performance testing and comparison, with three models in each category. The benchmarking comparison models are shown in Table 6.

**Fig. 9:**
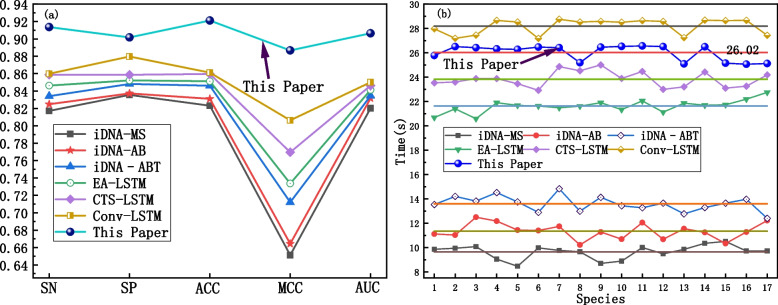
The prediction results of each comparative model. **a** Dot-line plot of evaluation index. **b** Dot-line plot of computation time

To ensure the fairness of these comparisons, we utilized the same testing set to operate each model on identical hardware and software systems. We randomly selected 500 data samples from each of the 17 species in the dataset to create a distinct database. Subsequently, individual training and testing were conducted for each model. For more detailed information, such as the parameters setting of each model, please refer to the relevant literature. The performance evaluation metrics and the average computation time of each comparison model are illustrated in Fig. 9.

**Fig. 10:**
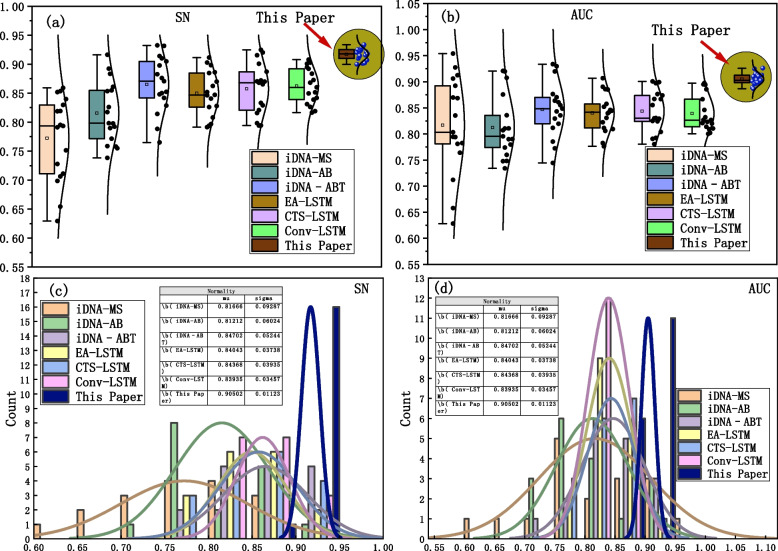
Prediction results of each comparative model in 17 species. **a** Distribution of SN for each model. **b** Distribution of AUC for each model. **c** Statistics of SN for each model. **d** Statistics of AUC for each model

It can be observed that the comprehensive performance of the iDNA-MS, iDNA-AB, iDNA-ABT, EA-LSTM, CTS-LSTM, and Conv-LSTM models gradually improves in terms of SN, SP and ACC, as shown in Fig. 9a. However, the Multi2-Con-CAPSO-LSTM model surpasses the others in all the metrics, demonstrating an obvious advantage. As shown in Fig. 9b, the computation time of the models is generally comparable across the 17 species. The iDNA-MS, iDNA-AB, and iDNA-ABT models exhibit shorter computation times, whereas the EA-LSTM, CTS-LSTM, Conv-LSTM, and the proposed Multi2-Con-CAPSO-LSTM model require relatively longer computation times. The average computation time of the Multi2-Con-CAPSO-LSTM model is 26.02 s, which is considered within an acceptable time range. The statistics and distribution of SN and AUC for each model across the 17 species are shown in Fig. 10.

From the distribution of evaluation metrics, both SN and AUC, the proposed model in the study ranks in the highest range, indicating its excellent predictive performance, as shown in Fig. 10a and b. According to the statistical results depicted in Fig. 10c and d, there are 16 data points near 0.94 for the SN metric, and 1 data point close to 0.91. In addition, for the AUC metric, there are 11 data points nearing 0.93, and 6 data points around 0.90. In summary, when compared to other benchmark models, the Multi2-Con-CAPSO-LSTM model demonstrates superior predictive performance. The average values of the evaluation metrics for each model across the 17 species are shown in Table 7.

**Table 7:** The statistical values of the evaluation metrics for each prediction model

Index	iDNA-MS	iDNA-AB	iDNA–ABT	EA-LSTM	CTS-LSTM	Conv-LSTM	This Paper
SN	0.8172	0.8248	0.8342	0.8463	0.8589	0.8600	0.9135
SP	0.8356	0.8373	0.8481	0.8522	0.8587	0.8798	0.9017
ACC	0.8231	0.8311	0.8461	0.8513	0.8597	0.8612	0.9211
MCC	0.6513	0.6644	0.7121	0.7337	0.7696	0.8064	0.8867
AUC	0.8203	0.8318	0.8346	0.8413	0.8463	0.8501	0.9064

The statistical data presented in Table 5 also demonstrate the significant advantage of the Multi2-Con-CAPSO-LSTM model, which can be attributed to the following three factors: (1) The hybrid encoding method of DNA sequences supplies the model with multivariate data. The model combines three types of temporal sequence encoding and three types of gene feature encoding for DNA sequences, providing reliable and multivariate foundational data for subsequent feature extraction. (2) The CNN and CAPSO method provide assurance for feature selection and parameter selection in the model. Through convolutional operations, the model transforms multivariate data into feature matrices, encapsulating temporal, spatial, and biochemical information. At the same time, the CAPSO method provides a solution for obtaining optimal parameters. (3) The LSTM network fully capitalizes on the long-term and short-term information within the DNA sequences, bolstering the prediction speed of the model. Given the pre- and post-relationships between DNA sequence methylation and the sequence itself, LSTM can effectively utilize these contextual relationships, thereby enhancing performance. Comparative experiments have shown that the Multi2-Con-CAPSO-LSTM model exhibits significant advantages in terms of sensitivity, specificity, accuracy, and correlation, compared to other benchmark models. Whether general methylation predictors or improved prediction methods, the prediction performance of the model in this paper is superior.

## Conclusions

In the paper, we propose a hybrid integrated learning model called Multi2-Con-CAPSO-LSTM. Firstly, compared to other models, Multi2-Con-CAPSO-LSTM demonstrates superior predictive performance. Secondly, through experiments conducted on 17 species with various methylation types, including 4mC, 5hmC, and 6 mA, the Multi2-Con-CAPSO-LSTM model has demonstrated excellent overall performance and effectively predicts multiple types of DNA methylation. Thirdly, as a machine learning-based DNA methylation model, Multi2-Con-CAPSO-LSTM integrates the positional information, physiological properties, and biological information of the DNA sequence, which ensures its good performance. The Multi2-Con-CAPSO-LSTM model provides a valuable reference for many disciplines such as biology, computer science, chemistry, and medicine. It covers a wide range of research areas including sequence alignment, genetic evolution, time series analysis, and structure–activity relationship studies. Although the proposed model in the study has achieved satisfactory results, there are still many challenges to address when facing the large-scale DNA methylation data. There are many issues that require further exploration. For example, how to improve the time and space complexity of machine learning methods, and how to design encoding methods that can extract as much global information from DNA sequences as possible. Currently, there are still some controversies in the research on biological sequences based on time series analysis methods. In future research, we will continue to delve into the key issues of the cross-disciplinary study of time series and biological sequences, aiming to make modest yet meaningful contributions to the integration and development of these two fields.

## Data Availability

The datasets generated and/or analysed during the current study are available free of charge at GitHub. (https://github.com/gnnumsli/Wu-Yan-DNA-methylation).
